# Dr. Francis Marion Perine

**Published:** 1903-08

**Authors:** 


### OBITUARY.

---

Dr. Francis Marion Perine, of Dansville, N. Y., died at his home in that village, July 11, 1903, aged 72 years. He was a graduate of the University of Buffalo, class of 1855, and practised his profession in Dansville and vicinity for nearly 50 years. He held the office of coroner for 21 years, and was president of the village at one time. He is survived by his wife.

